# High-dimensional entanglement certification

**DOI:** 10.1038/srep27637

**Published:** 2016-06-17

**Authors:** Zixin Huang, Lorenzo Maccone, Akib Karim, Chiara Macchiavello, Robert J. Chapman, Alberto Peruzzo

**Affiliations:** 1 Quantum Photonics Laboratory, School of Engineering, RMIT University, Melbourne, Victoria 3000, Australia; 2 School of Physics, University of Sydney, NSW 2006, Australia; 3 Dip. Fisica and INFN Sez. Pavia, University of Pavia, via Bassi 6, I-27100 Pavia, Italy

## Abstract

Quantum entanglement is the ability of joint quantum systems to possess global properties (correlation among systems) even when subsystems have no definite individual property. Whilst the 2-dimensional (qubit) case is well-understood, currently, tools to characterise entanglement in high dimensions are limited. We experimentally demonstrate a new procedure for entanglement certification that is suitable for large systems, based entirely on information-theoretics. It scales more efficiently than Bell’s inequality and entanglement witness. The method we developed works for arbitrarily large system dimension *d* and employs only two local measurements of complementary properties. This procedure can also certify whether the system is maximally entangled. We illustrate the protocol for families of bipartite states of qudits with dimension up to 32 composed of polarisation-entangled photon pairs.

As the dimension of investigated systems increases, it becomes more complicated to demonstrate their quantum effects^1^,^2^,^3^. Indeed, a full characterization (quantum tomography) becomes practically impossible already for systems of rather small dimensionality^4^,^5^. It is therefore important to explore new avenues to prove the presence of such effects, e.g. entanglement, for arbitrary dimensions. High-dimensional entangled states offer a larger code space, attracting interests for quantum key distribution^6^, teleportation^7^ and security-enhanced quantum cryptography^8^,^9^.

In this context, proving that one has achieved entanglement (entanglement certification) and detecting entanglement are different primitives. Indeed, entanglement detection methods^10^,^11^,^12^,^13^,^14^ must be as sensitive as possible and must be able to detect the largest possible class of entangled states. Often such methods are inapplicable to large system dimensions or scale poorly with increasing dimension as they entail increasingly complicated measurements and data analysis. Entanglement detection such as witness operators, for instance, typically requires a number of local measurement settings that scales linearly in *d*^15^. In contrast, entanglement certification refers to the fact that one has simply to prove that the system is entangled. To do entanglement certification, one can optimize the method to the specific entangled state that one is producing. It must fulfill different requirements: it must be as robust and simple as possible. In other words a good method for entanglement detection can also work for entanglement certification, but not vice versa.

Here, we present an entanglement certification procedure that is extremely simple to implement (the measurement of two local observables^16^,^17^ suffices), is compatible with current state-of-the-art experimental techniques, and can be easily scaled up to arbitrary dimension. In addition to certifying the production of entangled states, our procedure can also certify the production of maximal entanglement. To prove its simplicity, we present an experimental test that uses entangled systems with dimension up to *d* = 32, constructed by suitably grouping couples of entangled photon pairs. In the general case, for a two-qudit experiment with arbitrary *d*, two measures would still be sufficient to implement our method, but not sufficient for tomography or other entanglement detection methods. To certify the presence of entanglement, one has only to calculate the (classical) correlations among the measurement outcomes of the two observables, for example, through their mutual information. If such correlations are larger than some threshold, the state is guaranteed to be entangled (Fig. 1). If they attain their maximum value, the state must be maximally entangled.

If *A* and *C* are complementary properties, the knowledge of *A* gives no information on *C* and vice versa. This happens whenever |〈*a*|*c*〉|^2^ = 1/*d*, for all |*a*〉 and |*c*〉 eigenstates of the observables *A* and *C*, where *d* is the Hilbert space dimension. This is equivalent to two sets of mutually unbiased bases (MUB’s). Consider the two-qubit maximally entangled state |Φ^+^〉 ∝ |00〉 + |11〉; it clearly has maximal correlation among results for the observables with eigenstates |0〉, |1〉: both qubits have the same value. This state has also maximal correlation among results for a complementary observable with eigenvalues 
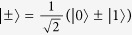
, since it can be written as |Φ^+^〉 ∝ |+ +〉 + |− −〉. Thus, the mutual information between measurement outcomes on the two qubits is one bit per observable, summing to two bits. If one has only separable states, the sum cannot be larger than one: for example, the classically correlated state *ρ* ∝ |00〉 〈00| + |11〉 〈11| has one bit of mutual information for the outcomes of the first observable, but zero bits for the outcomes of the second.

Starting from the theoretical suggestions of^18^, we extend the method from the qubit (*d* = 2) case to arbitrary high dimensions. The proposed mechanism to certify entanglement thus uses the following procedure:

1. Identify two bipartite complementary observables *AB* and *CD*, where *A*, *C* are system 1 observables and *B*, *D* are system 2 observables (Fig. 1a).
2. Measure the statistics of the outcomes of the two observables: local measurements on the two systems suffice.
3. These measurements return the joint probabilities *p*(*a*_*o*_, *b*_*o*_) of obtaining outcome *a*_*o*_ on system 1 for *A* and *b*_*o*_ on system 2 for *B*, and *p*(*c*_*o*_, *d*_*o*_) of obtaining outcome *c*_*o*_ on system 1 and *d*_*o*_ on system 2. Use these results to calculate the mutual information *I*_*AB*_ among measurement results for *AB* and *I*_*CD*_ among results for *CD*, with 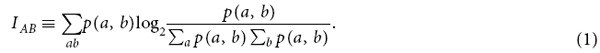
4. Certification: if *I*_*AB*_ + *I*_*CD*_ > log_2_ *d*, the two systems are entangled; if *I*_*AB*_ + *I*_*CD*_ = 2 log_2_ *d*, the two systems are maximally entangled.

The proof of these statements, based on Maassen and Uffink’s entropic uncertainty relation^19^ is given in^18^ (see also^20^,^21^,^22^).

In regards to the choice of observables, for pure states, the obvious choice would be the Schmidt bases and their respective Fourier bases. Whilst for mixed states, one can diagonalise the density matrix, identify the eigenvector with the largest weight and use the Schmidt basis (and its respective Fourier bases). Alternatively, one can choose the bases that diagonalises the reduced density matrices. While there is no guarantee that these choices will allow one to implement the procedure, they are the ones that may uncover the most correlations.

This method is simple to implement for systems of any dimensionality: it only entails independent measurements of two local observables on the two systems. Moreover, it is robust since, although one can optimize the choice of observables to maximize the sum of mutual information, the systems are guaranteed to be entangled if the above conditions are satisfied for *any* couple of complementary observables. It is interesting to note that, coherently performing sequential complementary measurements on the same system may generate the entanglement itself^23^.

To date, the most prominent way of producing higher dimensional entangled systems is via the orbital angular momentum degree of freedom of a photon^6^,^24^,^25^; schemes to produce three-level entangled states in trapped ions have also been proposed^26^,^27^. Our method, however, works for any *d*-dimensional system as long as the appropriate measurements can be performed.

Based on the theoretical work by Collins *et al*., other recent experiments^25^,^28^ have studied higher dimensional entangled system via generalised Bell’s inequalities, where the correlations between the two measurement settings on each qudit system are studied. The violation of Bell’s inequality is usually discussed in terms of quantum non-locality. In this context, the framework of hidden variable theories is in general specified by the 4 measurement settings, each with *d* outcomes, therefore needing *d*^4^ joint probabilites^29^ to describe the system globally. In this case^25^,^28^,^29^, examine two settings on each system, and how the observables on one system are correlated with the two observables on the second system, requiring a total of 2*d* × 2*d* joint outcomes. On the other hand, our method is based on entropic relations, requiring only two measurement settings, and is completely specified by 2*d*^2^ probabilities. Similarly to^28^, we used ensembles of individual entangled photon pairs to construct a higher dimensional state. All the subsystems that compose this state do not exist at the same time, but that is irrelevant to our current aims.

To demonstrate the method in practice, we performed an experiment using high-dimensional entangled systems. We certify the presence of entanglement for various families of states, by measuring pairs of complementary observables. These families are obtained by appropriately grouping couples of polarisation entangled photonic qubits, mapping qudits onto qubits:
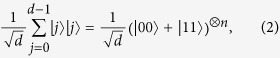
where this mapping is obtained by expressing *j* in binary notation and rearranging the qubits in such a way that the ones placed in odd positions in the tensor products on the right hand side are assigned to system 1 while the ones in even positions to system 2. For example, a *d* = 4 maximally entangled state can be expressed as (|00〉 + |11〉 + |22〉 + |33〉)/2 ≡ (|00 00〉 + |01 01〉 + |10 10〉 + |11 11〉)/2 ≡ (|0〉_*α*_|0〉_*β*_ + |1〉_*α*_|1〉_*β*_) ⊗ (|0〉_*γ*_|0〉_*δ*_ + |1〉_*γ*_|1〉_*δ*_)/2, where qubits labeled *α* and *γ* belong to system 1, while *β* and *δ* to system 2. It would also be extremely interesting to show that one could coherently manipulate the *n* entangled pairs locally, though that is not necessary to implement our method (which is also one of the main strengths of the method). An analogous mapping applies to the mixed states:
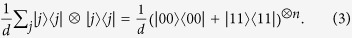


Regarding the necessary measurements, the observable *AB* corresponds to *σ*_*z*_, namely the computational basis {|0〉, |1〉} for each qubit. As to the observable *CD*, we will consider two possibilities: the Fourier basis and the *σ*_*x*_ basis. The Fourier basis is defined as
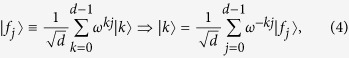
with *ω* ≡ exp(2*πi*/*d*). It can be expressed as tensor products of single-qubit states (see Supplementary Material).

The Fourier basis in arbitrary dimension identifies an observable complementary to the computational basis. However, in the case considered here, there are complementary bases that are simpler to access experimentally, namely the bases where one measures *σ*_*x*_ or *σ*_*y*_ on each qubit. We consider *σ*_*x*_ here. The *σ*_*x*_ basis is given by |*c*_*k*_〉 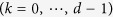 obtained by expressing the binary digits of *k* in the {+, −} basis, i.e.
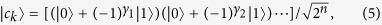
where 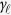 are the bits of the number *k*. The *σ*_*x*_ basis is complementary to the computational basis, since |〈*j*|*c*_*k*_〉|^2^ = 1/2^*n*^ = 1/*d* for all *j*, *k*.

We tested our entanglement certification procedure on several families of states. These particular states that we consider are arbitrarily chosen for their simplicity. For the specific examples, the entanglement of the *d*-dimensional entangled pair is exactly the same as the one obtained from consecutive entangled two-photon states. This happens only when *d* is a power of two, though the method works for any *d*. The first is
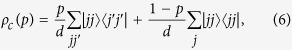
which mixes the maximally entangled state of equation (2) with the state in equation (3) with classical correlation only on the computational basis, the above state is always entangled for *p* ≠ 0.

The experimental test of this prediction is presented in Fig. 2, red circles (the markers are the data points and the connecting lines are the expected curves fitted to density matrices from quantum state tomography).

The second family of states we consider are the Werner states: a mixture of a maximally entangled and a maximally mixed state,
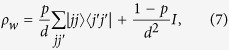
where *I* is the identity *d*^2^ × *d*^2^ matrix. These states are entangled for *p* > 1/(*d* + 1) (e.g.^30^). Their mutual information is experimentally determined as the green triangles in Fig. 2.

The third family of states is
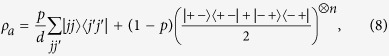
where 
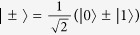
. The experimental results are plotted in Fig. 2 as blue stars (when the *CD* observable is the *σ*_*x*_ basis |*c*_*k*_〉) and as purple diamonds (when *CD* is the Fourier basis |*f*_*j*_〉). Measurements in the Fourier basis and in the *σ*_*x*_ basis are performed for *d* = 4 and 8 only, due to the large number of projections required for higher dimensions where temporal phase instability would significantly affect the measurements. This state shows the difference between the complementary bases: its form implies that correlations are greater in the *σ*_*x*_ than the Fourier basis.

The entanglement certification method can be modified by using a different measure of correlations. Indeed, instead of the mutual information, one can also measure correlations with the Pearson coefficient
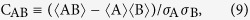
where 〈*X*〉 is the expectation value of *X* and 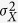 its variance. It measures the linear correlation between the outcomes of two observables *A* and *B*, and takes values 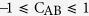. It is conjectured^18^ that if C_AB_ + C_CD_ > 1, then the state is entangled. Moreover, it is known that if C_AB_ + C_CD_ = 2, the state is maximally entangled. This may be helpful in high-noise scenarios, since it allows for the certification of a larger class of entangled states. The Pearson correlation coefficient seems to be more robust against imbalanced probabilities or decoherence in the experiment (see Fig. 3), and numerical simulations suggest that it is more effective in detecting entanglement^18^. So, in addition to the sum of mutual information *I*_*AB*_ + *I*_*CD*_, we can use C_AB_ + C_CD_ as an alternative way of certifying entanglement (modulo a conjecture).

We consider the two-qubit states
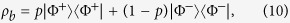

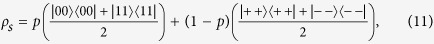
and the Werner state *ρ*_*w*_ of Eq. (7) with *d* = 2. In (10), 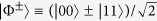. The state *ρ*_*b*_ is entangled for *p* ≠ 1/2, whereas *ρ*_*s*_ is always separable and has zero discord only for *p* = 0, 1. For two-qubit states, the *σ*_*x*_ basis |*c*_*k*_〉 coincides with the Fourier basis |*f*_*j*_〉, so the two possible observables *CD* we used above coincide here.

However, we preferred working with mutual information since the fact that the Pearson coefficient can be used to certify entanglement is still a conjecture, whereas it has been rigorously proved for the sum of mutual information.

Whilst it is true that the standard entanglement witness
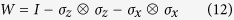
out-performs our method for entanglement dection for the (*d* = 2 case) Werner state (detects entanglement at *p* ≥ 0.5), this is not true for arbitrary dimensions in general.

Finally, also comparing this against Bell’s inequalities using the Werner state, our method using mutual information with two complementary observables (computational and *σ*_*x*_ basis), for *d* = 2, the threshold of log_2_(*d*) is surpassed at *p* ≈ 0.78. However, if we increase this to three observables (indeed one can, and in this case we consider the *σ*_*y*_ basis), the entanglement is detected at *p* ≈ 0.65 (see supplementary material of^18^), which outperforms Bell’s inequalities at 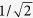
11. Also, in the limit of large *d*, our method (see Eq. (S7) in the Supplementary Material) also out-performs the threshold in Bell’s inequality violation, given in^29^.

In summary, we have presented an entanglement certification method that is suitable for large dimensional systems. To our knowledge, there are no such methods that are as efficient to implement on a 32-dimensional system such as the one we experimentally study here. We have shown that entangled states are more correlated on the outcomes of complementary observables. One may think that this property is shared by other types of quantum correlations such as quantum discord^31^ but, surprisingly, this is not the case, at least when the correlations are gauged through mutual information. Indeed, the separable state *ρ*_*s*_ has highest *I*_*AB*_ + *I*_*CD*_ for *p* = 0, 1 (Fig. 3), the values for which its discord is null. In contrast, *I*_*AB*_ + *I*_*CD*_ is lowest for *p* = 1/2 which is where such state has highest discord. This unexpected property is lost when the correlation is gauged with the Pearson coefficient, as C_AB_ + C_CD_ = 1 for all values of *p*.

## Methods

**Figure 4:**
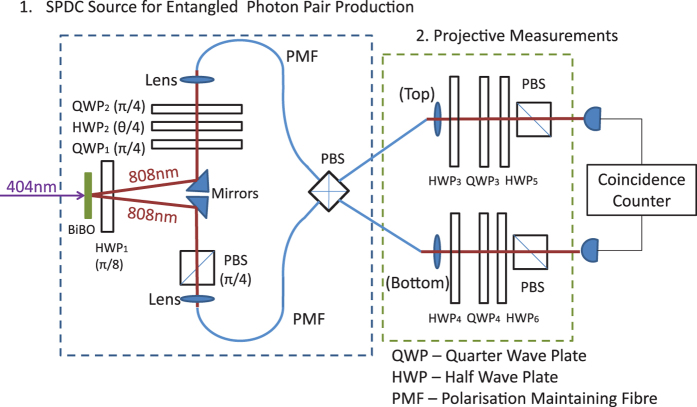
Scheme for polarisation entangled source. Type-I SPDC source is used to generate pairs of horizontally polarised single photons. HWP_1_ rotates |HH〉 to ++〉, 
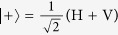
. The top arm experiences a polarisation phase shift, and the state of the bottom arm is purified and matched to the top arm by the PBS rotated by *π*/4 radians, which transmits |+〉. The photons are collected into polarisation maintaining fibres (PMF) and are incident on the two input faces of a PBS, which transmits H and reflects V. This prepares the state 
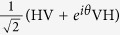
 when measuring in the coincidence basis. Standard tomography set up projects the state onto different bases for measurements. See text for further details.

In order to generate polarisation-entangled photon pairs, a set up similar to^32^ and identical to^33^ is used. We use the polarisation encoding where |H〉 ≡ |0〉 and |V〉 ≡ |1〉. The set up is shown in Fig. 4. A type-I nonlinear crystal (BiBO) is pumped with a vertically polarised cw laser (404 nm) at 80 mW, generating pairs of horizontally polarised single photons (808 nm). The photons are spectrally filtered using 3 nm top-hat filteres centred on 808 nm. The first half way plate HWP_1_ (optics axis set to *π*/8 radians) rotates the output photon pair from |HH〉 to |+ +〉, where 
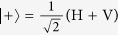
. The polarisation in the top arm is matched and purified to that of the bottom arm using a polarisation beam splitter (PBS) rotated by *π*/4 radians, which transmits |+〉.

The unitary operation realised by the sequence of wave plates QWP_1_ + HWP_2_(*θ*) + QWP_2_, as a function *θ* of the HWP_2_ angle, applies a phase to photon in V state, effectively that of a phase gate: 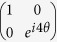. Denoting the input photon in the top (bottom) arm with subscript 1 (2), the two-photon state collected into the polarisation mainting fibre (PMF) is therefore 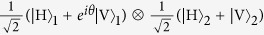.

We use a silicon avalanche single photon counter and measure in coincidence with a time window of 2.5 ns. At the fibre coupled PBS, H is transmitted and V is reflected. After the fibre PBS crystal, the top (bottom) arm fibre contains the components (V_1_ and  H_2_) (H_1_ and  *e*^*iθ*^ V_2_). However, the design of the fibre PBS is such that the output coupler flips the polarisation state in the bottom arm. Without this flip, the registered coincidence will be either *e*^*iθ*^ |V〉_1_ |V〉_2_ or |H〉_1_ |H〉_2_. After the flip the detector will register either *e*^*iθ*^ |V〉_1_ |H〉_2_ or |H〉_2_ |V〉_1_, and these two components are made spatially indistinguishable by adjusting the position of the fibre, giving the post-selected state 
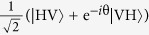
. HWP_3_ and HWP_4_ apply the appropriate bit flip operations on each single qubit. QWP_3_ + HWP_5_ and QWP_4_ + HWP_6_ along with the final two PBSs project each photon onto any polarisation state.

The polarisation maintaining fibres have a beat length (H and V delayed with respect to each other by 2*π*) of 24 mm; the coherence length of the photon pairs is 250 *μ*m (extracted from a Hong-Ou-Mandel dip of 99% visibility), which means that if uncompensated, 1.0 m of fibre will spatially separate the H and V components of a photon out of coherence, turning a Bell state 
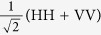
 into the classically correlated mixture 
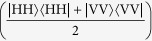
. When producing a Bell state, this spatial decoherence was compensated by crossing the slow axis of the PMF and tuning the stage position of the fibre (and vice versa when the mixed state is required).

## Additional Information

**How to cite this article**: Huang, Z. *et al*. High-dimensional entanglement certification. *Sci. Rep.* **6**, 27637; doi: 10.1038/srep27637 (2016).

## Supplementary Material

Supplementary Information

## Figures and Tables

**Figure 1:**
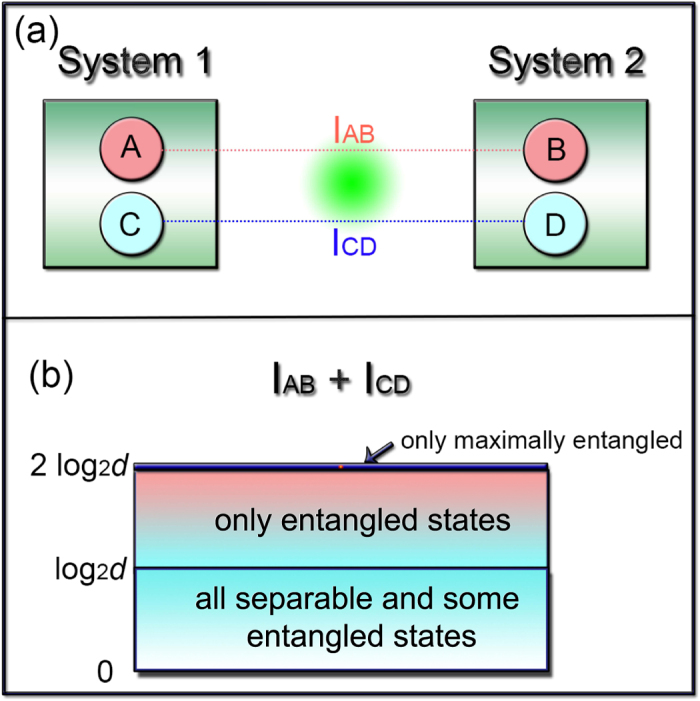
Illustration of the procedure. (**a**) Measure two complementary observables: *AB* and *CD*, where *A*, *C* are observables for system 1 and *C*, *D* for system 2. Then calculate the mutual information *I*_*AB*_ and *I*_*CD*_ of their outcomes. (**b**) If the value of the sum *I*_*AB*_ + *I*_*CD*_ is larger than log_2_(*d*) bits (*d* being the system dimension), then the two systems are certified to be entangled. If the value of the sum is 2 log_2_(*d*) bits, they are certified to be maximally entangled.

**Figure 2:**
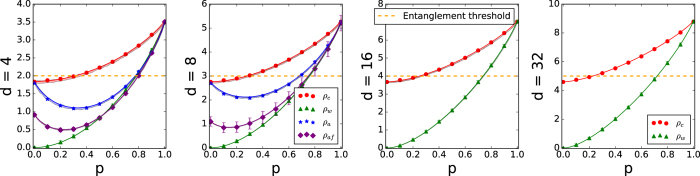
Experimental test of the method for *d*-dimensional systems. The plots refer to the experimentally determined sum of mutual information *I*_*AB*_ + *I*_*CD*_ for *ρ*_*c*_ of equation (6) (red circles), *ρ*_*w*_ of equation (7) (green triangles), *ρ*_*a*_ of equation (8) (blue stars when *CD* is the *σ*_*x*_ observable, and purple diamonds when *CD* is the Fourier basis). The points that fall above the entanglement threshold (orange dashed line) refer to states that are *certified* to be entangled. Taking into account the confidence interval of the measurement outcome, the state was maximally entangled if the maximum sum *I*_*AB*_ + *I*_*CD*_ = 2 log_2_ *d* is achieved. The bands are a Monte Carlo simulation, taking into account realistic experimental conditions (e.g. phase inaccuracy due to the wave plates used in state tuning); for *d* = 32 they are narrower than the fit. The error bars represent bounds for 2 standard deviations (95% confidence), for most of the data points the error bars are smaller than the markers.

**Figure 3:**
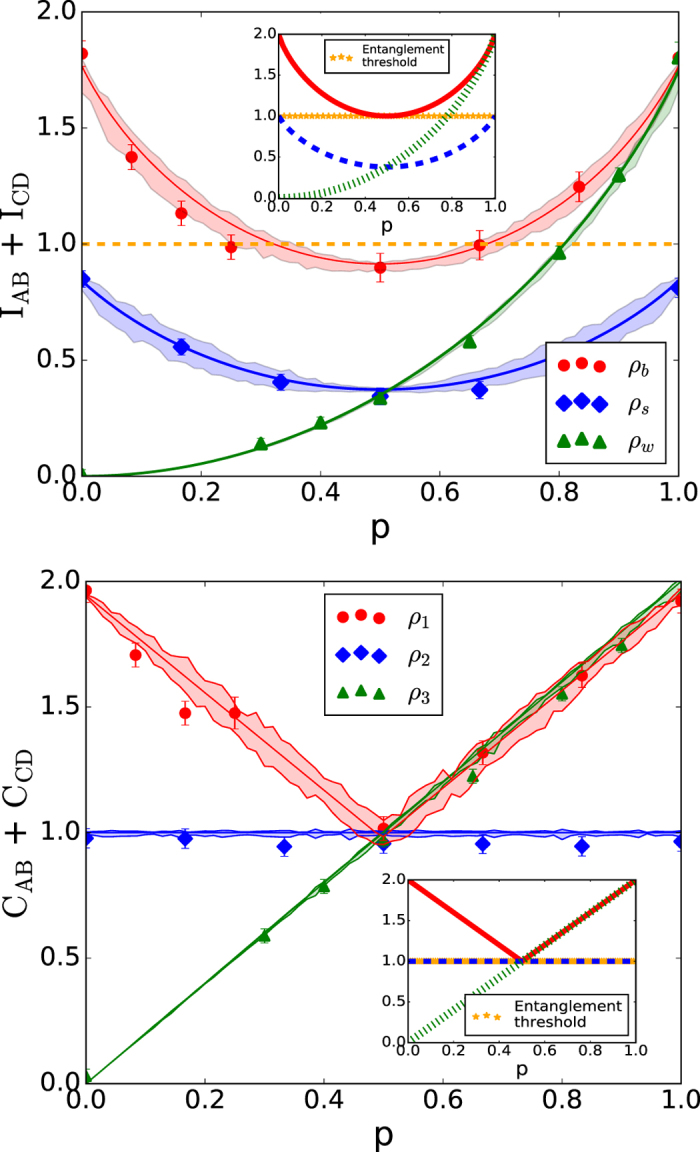
Experimental comparison between mutual information (above) and Pearson coefficient (below). The insets show the theoretical curves. The data points refer to *ρ*_*b*_ of equation (10) (red circles), *ρ*_*s*_ of equation (11) (blue diamonds), *ρ*_*w*_ of equation (7) (green triangles) for *d* = 2. The states whose data points are strictly above the entanglement threshold (*I*_*AB*_ + *I*_*CD*_ = 1 for the upper graph and *C*_*AB*_ + *C*_*CD*_ = 1 for the lower graph, conjectured) are certified to be entangled. In the lower graph this threshold overlaps with the fit for *ρ*_*s*_, which is then not entangled, as expected. The markers represent the data points, the lines represent a fit from reconstructed density matrices, and the bands are a Monte Carlo simulation, taking into account realistic experimental conditions (eg. phase inaccuracy due to the wave plates used in state tuning). The error bars represent bounds for 2 standard deviations. It is evident that the experimental results are in good agreement with the theoretical values.
