# Supplementary material for: High-dimensional entanglement certification
Source: Sci Rep. 2016 Jun 17;6:27637. doi: 10.1038/srep27637 (PMC4911610; doi:10.1038/srep27637)
Supplement: Supplementary Information [file srep27637-s1.pdf]

High-dimensional entanglement certification
Supplemental Materials

Zixin Huang[1], Lorenzo Maccone[2], Akib Karim[1], Chiara Macchiavello[2], Robert J. Chapman[1], Alberto Peruzzo[1]

1. Quantum Photonics Laboratory, School of Electrical and Computer Engineering, RMIT University, Melbourne, Australia and School of Physics, University of Sydney, NSW 2006, Australia.

2. Dip. Fisica and INFN Sez. Pavia, University of Pavia, via Bassi 6, I-27100 Pavia, Italy

The Fourier basis

The Fourier basis can be written as single-qubit tensor products in the following way:

$$|f_j\rangle = \frac{1}{\sqrt{d}} \bigotimes_{k=0}^{n-1} (|0\rangle + \omega^{j2^k}|1\rangle), \tag{S1}$$

by expressing k in Eq.(4) in binary form. Using equation (4), the maximally entangled state in equation (2) is perfectly correlated (actually, anti-correlated) in this basis:

$$\tfrac{1}{\sqrt{d}}\textstyle\sum_j |j\rangle|j\rangle = \tfrac{1}{\sqrt{d}}\sum_k |f_k\rangle|f_{-k}\rangle. \tag{S2}$$

The σ_x basis

The maxiamlly entangled state $|00\rangle + |11\rangle = |{+}{+}\rangle + |{-}{-}\rangle$, the mapping (2) can be trivially applied to the σ_x basis, namely

$$\sum_j |j\rangle|j\rangle = (|++\rangle + |--\rangle)^{\otimes n} = \sum_k |c_k\rangle|c_k\rangle, \tag{S3}$$

showing that the maximally entangled state in equation (2) is maximally correlated also in the σ_x basis.

Analytical expressions for mutual information

On the state ρ_c, the joint probabilities for the computational basis is $p(a_o, b_o) = \delta_{a_o b_o}/d$ with δ_{ab} the Kronecker delta. So the mutual information for the computational basis is $I_{AB} = \log_2 d$ as expected: there is perfect correlation on such basis in both terms of (6). Writing ρ_c in the σ_x basis, we can calculate the joint probabilities for it as $p(c_o, d_o) = \delta_{c_o,d_o} p/d + (1-p)/d^2$ (i.e. there is still maximal correlation on the entangled part of ρ_c, while there is no correlation on the rest). Whence we can calculate I_{CD} and find

$$I_{AB} + I_{CD} = \log_2 d + \tag{S4}$$
$$\tfrac{(1-p)(d-1)}{d}\log_2(1-p) + \tfrac{1+(d-1)p}{d}\log_2(1+(d-1)p).$$

(The same result is obtained also considering the Fourier basis for CD instead of the σ_x basis.)

Consider now the Werner state ρ_w. The joint probabilities for the two complementary observables (computational and Fourier bases) are respectively

$$p(a_o, b_o) = p/d\delta_{a_o b_o} + (1-p)/d^2\ , \tag{S5}$$
$$p(c_o, d_o) = p/d\delta_{a_o,-b_o} + (1-p)/d^2 \tag{S6}$$
$$\Rightarrow I_{AB} + I_{CD} = 2I_{AB} = \tag{S7}$$
$$2\left[\tfrac{1+(d-1)p}{d}\log_2[1+(d-1)p] + \tfrac{(1-p)(d-1)}{d}\log_2(1-p)\right],$$

which is experimentally determined as the green triangles in Fig.2. The mappings (S2) and (S3), and the fact that $\mathbb{1}$ remains unchanged in any basis implies that $I_{AB} = I_{CD}$ is the same for all the observables considered here (computational, Fourier and σ_x). Given the symmetry of ρ_w, our method is able to certify entanglement only for highly-entangled Werner states.

Density matrices

The states used in constructing the family of states as in Eq. (6), (7) and (8) are primarily the Bell states $\frac{1}{\sqrt{2}}(|01\rangle + |10\rangle\,|)$, $\frac{1}{\sqrt{2}}(|01\rangle - |10\rangle\,|)$ and the decohered, classically correlated state $\frac{|01\rangle\langle 01| + |10\rangle\langle 10|}{2}$. All the others can be accessed by applying the appropriate local unitary transformation. The density matrices obtained by performing quantum state tomography are shown in Fig.S1,S2 and S3. The fidelities are given in the captions.

$d = 2$ states generation

ρ_b is generated by averaging measurements for $|\Phi^+\rangle\langle\Phi^+|$ with phases applied to a single qubit. ρ_s is generated by decohering the Bell-state $\frac{1}{\sqrt{2}}(|00\rangle + e^{i\phi}|11\rangle)$ to $\frac{|00\rangle\langle 00| + |11\rangle\langle 11|}{2}$ (See Methods), followed by averaging with the appropriate partial bit flip ($\sigma_x \otimes \sigma_x$) applied to both qubits. For each value of p, 40 iterations were run, the upper and lower bounds of the bands are given by the mean of the 40 points $\pm$ 2 standard deviations respectively. The typical count rates were in the order of 800 coincidences/sec, with accidentals subtracted; for each projective measurement, a 10-second integration

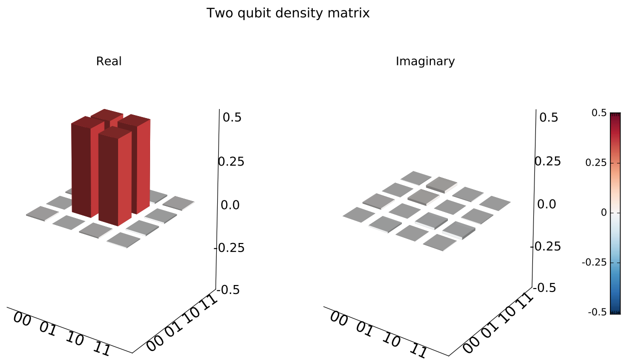

FIG. S1. Density matrix for $\frac{1}{\sqrt{2}}(|01\rangle + |10\rangle\,|)$, fidelity $= 97.6\%$ after maximum likelihood [1]

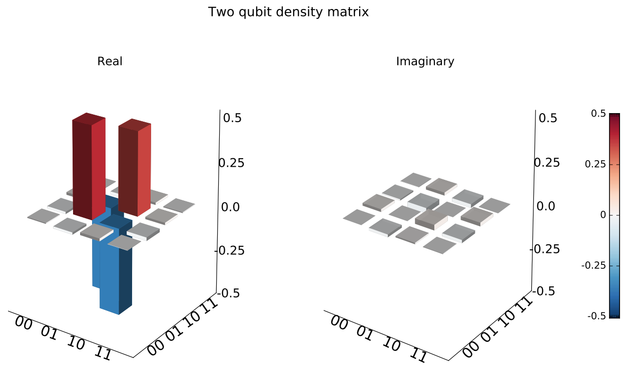

FIG. S2. Density matrix for $\frac{1}{\sqrt{2}}(|01\rangle - |10\rangle\,|)$, fidelity $= 97.8\%$ after maximum likelihood.

time was used such that error due to Poissonian noise would account for $\leq 1\%$ of the measured probability.

ρ_w is made by measuring $|\Phi^+\rangle\langle\Phi^+|$ and $(\mathbb{I}/4)$ in a time-sharing fashion. All of the states in $d > 2$ are generated by combining pure states measurements.

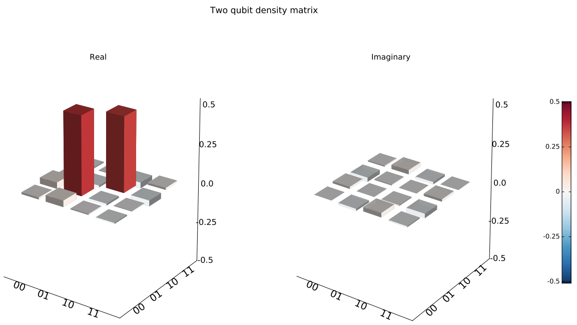

FIG. S3. Density matrix for $\frac{|01\rangle\langle 01| + |10\rangle\langle 10|}{2}$, fidelity $= 97.6\%$ after maximum likelihood.

DATA PROCESSING AND ERROR ANALYSIS

Measurement in each MUB involves projecting the state onto a given basis. For each basis projection onto ρ_i, the counts for that projection is normalised with respect to the normalisation factor ($\mathrm{N_{tot}}$), given : $\mathrm{N_{tot}} = \langle 00|\,\rho_i\,|00\rangle + \langle 01|\,\rho_i\,|01\rangle + \langle 10|\,\rho_i\,|10\rangle + \langle 11|\,\rho_i\,|11\rangle$

Each statistic was assumed to be a Poissonian process, with standard deviation $\Delta\mathrm{N_i} = \sqrt{\mathrm{N_i}}$. The error bars displays 2 stdev with the following method of analysis.

$\mathrm{p_i} = \mathrm{N_i}/\mathrm{N_{tot}}$, therefore $\Delta\mathrm{p_i} = \mathrm{p_i}(\frac{\Delta\mathrm{N_i}}{\mathrm{N_{tot}}} + \frac{\Delta\mathrm{N_{tot}}}{\mathrm{N_{tot}}})$ eg. with a total of 10000 counts, the following shows the basis vector, counts for the projection and probability:

$$\begin{pmatrix} HH \\ HV \\ VH \\ VV \end{pmatrix} \rightarrow \begin{pmatrix} 1000 \\ 4000 \\ 4500 \\ 500 \end{pmatrix} \rightarrow \begin{pmatrix} 0.10 \\ 0.40 \\ 0.45 \\ 0.05 \end{pmatrix} \tag{S8}$$

The Shannon entropy for qubit one is given by:

$$\mathrm{H(A) = -p(H)log2(p(H)) + p(V)log2p(V)} \tag{S9}$$

ie

$$\begin{aligned} \mathrm{H(A)} = &-((0.1+0.4)\mathrm{log2}(0.1+0.4) \\ &+(0.45+0.5)\mathrm{log2}(0.45+0.45) \end{aligned} \tag{S10}$$

$$\begin{aligned} \mathrm{H(A|B)} = &-((0.1)\mathrm{log2}(\frac{0.1}{0.1+0.45}) + (0.45)\mathrm{log2}(\frac{0.45}{0.1+0.45}) \\ &+(0.40)\mathrm{log2}(\frac{0.40}{0.40+0.05}) + (0.05)\mathrm{log2}(\frac{0.40}{0.40+0.05}) \end{aligned} \tag{S11}$$

The error in the probabilities are therefore:

$$\Delta p_i = \begin{pmatrix} 0.1(\frac{\sqrt{1000}}{1000} + \frac{\sqrt{10000}}{10000}) \\ 0.40(\frac{\sqrt{4000}}{4000} + \frac{\sqrt{10000}}{10000}) \\ 0.45(\frac{\sqrt{4500}}{4500} + \frac{\sqrt{10000}}{10000}) \\ 0.05(\frac{\sqrt{500}}{500} + \frac{\sqrt{10000}}{10000}) \end{pmatrix} \tag{S12}$$

$$\mathrm{I} = -\sum_i \mathrm{p_i log_2(p_i)} \tag{S13}$$

At each point displayed on the graphs in the main text, the error bars were calculated via error propagation. The uncertainty in information, ΔI then follows as:

$$\Delta\mathrm{I} = -\frac{1}{\ln(2)}\sum_{\mathrm{i}}(\Delta\mathrm{p_i}\ln(\mathrm{p_i}) + \mathrm{p_i}\Delta(\ln(\mathrm{p_i}))) \tag{S14}$$

$$= -\frac{1}{\ln(2)}\sum_{\mathrm{i}}(\Delta\mathrm{p_i}\ln(\mathrm{p_i}) + 1)) \tag{S15}$$

MAXIMAL MUB'S IN $d = 3$ AND 4

We used the MUB's from [2] to perform our calculations. Note that for d = 4, out of the four sets of MUB's provided, there is an error in that the two sets are identical. We amended this, and the ones we used are specified in Eq.(S19)

For $d = 3$:

$$\begin{aligned} \{|F3_a\rangle\} &= \{\frac{1}{3}[1,1,1], \frac{1}{3}[1,\omega,\omega^2], \frac{1}{3}[1,\omega^2,\omega]\} \\ \{|F3_b\rangle\} &= \{\frac{1}{3}[1,\omega,\omega], \frac{1}{3}[1,\omega^2,1], \frac{1}{3}[1,1,\omega^2]\} \\ \{|F3_c\rangle\} &= \{\frac{1}{3}[1,\omega^2,\omega^2], \frac{1}{3}[1,\omega,1], \frac{1}{3}[1,1,\omega]\} \end{aligned} \tag{S16}$$

where $\omega = e^{2\pi i/d}$. Together with the computational basis, these form the four sets of MUB's in $d = 3$.

For the computational and the Fourier basis ($\{|F3_a\rangle\}$), maximal correlation occur when the two systems are measured in these the same respective bases. For $\{|F3_b\rangle\}$ and $\{|F3_c\rangle\}$ however, there is zero correlation if both sides are measured in the same bases. To achieve maximum correlation, when system one is measured in $\{|F3_b\rangle\}$ ($\{|F3_c\rangle\}$), system two must be measured with a different set of MUB as defined in Eqn.(S17) ((S18)).

Comparing the mutual information for using just two MUB's (red dotted) and the full four MUB's, are given in Fig.S4.

$$\{\frac{1}{3}[\omega,1,1], \frac{1}{3}[1,1,\omega], \frac{1}{3}[1,\omega,1]\} \tag{S17}$$

$$\{\frac{1}{3}[\omega^2,1,1], \frac{1}{3}[1,1,\omega^2], \frac{1}{3}[1,\omega^2,1]\} \tag{S18}$$

For $d = 4$, we used the following bases:

$$\begin{aligned} \{|F4_a\rangle\} = \{&\frac{1}{2}[1,1,1,1,], \frac{1}{2}[1,1,-1,-1], \\ &\frac{1}{2}[1,-1,-1,1], \frac{1}{2}[1,-1,1,-1]\} \\ \{|F4_b\rangle\} = \{&\frac{1}{2}[1,-1,-i,-i], \frac{1}{2}[1,-1,i,i], \\ &\frac{1}{2}[1,1,i,-i], \frac{1}{2}[1,1,-i,i]\} \\ \{|F4_c\rangle\} = \{&\frac{1}{2}[1,-i,-i,-1], \frac{1}{2}[1,-i,i,1], \\ &\frac{1}{2}[1,i,i,-1], \frac{1}{2}[1,i,-i,1]\} \\ \{|F4_d\rangle\} = \{&\frac{1}{2}[1,-i,1,-i], \frac{1}{2}[1,-i,1,i], \\ &\frac{1}{2}[1,i,1,-i], \frac{1}{2}[1,i,-1,i]\} \end{aligned} \tag{S19}$$

Together with the computational basis, these form the five sets of MUB's in $d = 4$. The mutual information for

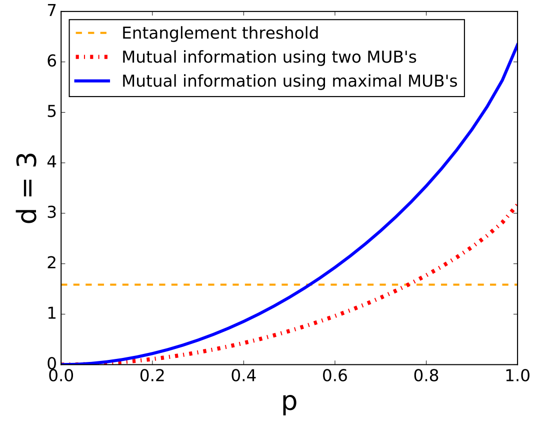

FIG. S4. Mutual information of the Werner state in Eqn (7) for $d = 3$ when measured in only two MUB's (red dotted line), or all five sets of MUB's (blue solid line)

the Werner state, when measured using two MUB's (red dotted) and the full five MUB's, are given in Fig.S5.

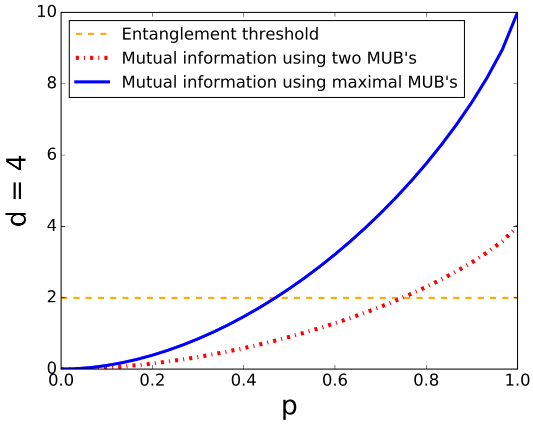

FIG. S5. Mutual information of the Werner state in Eqn (7) for $d = 4$ when measured in only two MUB's (red dotted line), or all five sets of MUB's (blue solid line)

REFERENCES

[1] James, D.F.V., Kwiat, P.G., Munro, W.J. & White, A.G., Measurement of qubits. *Phys.Rev.A* **64** 052312 (2001)

[2] Klappenecker, A. & Rötteler, M., Constructions of mutually unbiased bases. *Finite fields and applications* 137–144 (2004)
